# Probiotics treatment for Parkinson disease: a systematic review and meta-analysis of clinical trials

**DOI:** 10.18632/aging.204266

**Published:** 2022-09-09

**Authors:** Chien-Tai Hong, Jia-Hung Chen, Tsai-Wei Huang

**Affiliations:** 1 Department of Neurology, Shuang-Ho Hospital, Taipei Medical University, New Taipei City, Taiwan; 2 Department of Neurology, School of Medicine, College of Medicine, Taipei Medical University, Taipei, Taiwan; 3 School of Nursing, College of Nursing, Taipei Medical University, Taipei, Taiwan; 4 Department of Nursing, Wan Fang Hospital, Taipei Medical University, Taipei, Taiwan; 5 Center for Nursing and Healthcare Research in Clinical Practice Application, Wan Fang Hospital, Taipei Medical University, Taipei, Taiwan; 6 Cochrane Taiwan, Taipei Medical University, Taipei, Taiwan

**Keywords:** Parkinson disease, probiotics, constipation, meta-analysis

## Abstract

Background and aims: People with Parkinson disease (PwP) exhibit gut dysbiosis and considerable gastrointestinal (GI) symptoms. Probiotics, beneficial strains of microorganisms, supplement and optimize the intestinal environment and alleviate GI symptoms among elderly people. We conducted a systematic review and meta-analysis of clinical trials to investigate the effects of probiotics on PwP.

Methods: We searched the PubMed, Embase, and Cochrane Library databases. Major outcomes were the effects on GI symptoms, including bowel movement and stool characteristics. This study was registered with PROSPERO (CRD42021262036).

Results: Six randomized controlled trials (RCTs) and two open-label studies were included. Most of the probiotic regimens were based on Lactobacillus and Bifidobacterium. Six studies investigated the benefit of probiotics for GI symptoms, especially for PwP with functional constipation, and two RCTs assessed probiotics’ effect on systematic metabolism and inflammation. In the meta-analysis, probiotic treatment significantly increased the frequency of bowel movements among PwP (mean difference [MD]: 1.06 /week, 95% confidence interval [CI]: 0.61 to 1.51, p < 0.001, I^2^ = 40%). Additionally, probiotic treatment significantly normalized stool consistency (standard MD: 0.61, 95% CI = 0.31 to 0.91, p < 0.001, I^2^ = 0%).

Conclusions: Although the probiotic compositions varied, probiotic treatment significantly attenuated constipation for PwP and exhibited possible systematic effects on inflammation and metabolism. Given the tolerability of probiotics, the present meta-analysis may provide more consolidated evidence of the benefit of probiotics on constipation in PwP and a possible new therapeutic approach for disease modification.

## INTRODUCTION

Parkinson disease (PD) is a progressive neurodegenerative disorder which the majority of symptoms are caused by loss of dopaminergic neurons in the substantia nigra and pathology originate in the central nervous system (CNS) [1]. However, gastrointestinal (GI) symptoms, such as constipation and dyspepsia, are prevalent nonmotor symptoms (NMS) of PD [2]. The loss of neurons and the deposition of α-synuclein in the enteric nerve system, which represents the extra-CNS involvement of the disease pathology, are observed in people with PD (PwP) [3–5]. Furthermore, certain bowel conditions, including ulcerative colitis [6] and truncal vagotomy during gastrectomy [7, 8], affect the risk of PD.

Microorganisms, the primary inhabitants of the intestinal system, are also associated with PD. PwP tend to experience small intestinal bacterial overgrowth [9, 10], and their gut microbiota, the assemblage of microorganisms (bacteria, archaea, eukaryotes, and viruses) in the gut, is usually distinct from that of healthy controls [11]. In addition to PD, gut microbiota also plays a major role in Alzheimer disease [12] and aging [13, 14].

Some specific bacterial strains, such as *Bifidobacteria* and *Lactobacilli*, have been widely deemed to be health-promoting components of the gut microbiota [15]. Some pathobiontic bacteria, however, such as *Helicobacter hepaticus*, segmented filamentous bacteria, *Escherichia coli*, and *Enterococcus faecalis* may be pathologic and increase inflammation or tumorigenesis [16, 17]. The modulation of the gut microbiota can be achieved either by prebiotics (nondigestible food ingredients that, when consumed in sufficient amounts, selectively stimulate the growth and/or activity of one or a limited number of microbes in the colon), probiotics (live bacteria and yeasts that, when administered in appropriate doses, induce a beneficial effect on health), and synbiotics (the combination of both) [18]. The main purpose of microbiota modulation is to promote beneficial interactions between the gut microbiota and the host [19].

The application of prebiotics or probiotics increases the frequency of bowel movements and relieves constipation [20]. For PwP, constipation is a distressful NMS that is unresponsive to dopaminergic supplements. Laxative agents may temporarily attenuate its severity, but the response wanes rapidly [21]. The lack of proper management of constipation is a challenge, and constipation is strongly associated with a poor quality of life for PwP [22]. The etiology of constipation in PD is multifactorial, including the reduction of bowel movement frequency, less body motility, and an unhealthy gut environment [2]. A *Bifidobacteria* and *Lactobacilli*–based probiotic regimen increases bowel movement frequency and mitigates constipation among PwP [23, 24], but studies have been few and small in sample size, with consequent adverse effects on the results’ level of evidence. Furthermore, probiotics affect local inflammation, reduce pathogenic gut microorganism–related leaky gut syndrome [25, 26], and modulate metabolism [27]. These benefits may be associated with the pathogenesis of PD, leading to possible modification of disease progression.

Despite an understanding of the gut microbiota in PwP and the possible beneficial roles of probiotics, some essential points remain unclear, especially the beneficial and detrimental strains of bacteria for use in alleviating the symptoms of PD. PwP tend to have more *Lactobacilli* than controls do [11], and whether this difference is the cause or consequence of PD pathology is unknown. The present study reviewed the interventional clinical trials and conducted a meta-analysis of randomized controlled trials (RCTs) to investigate the effect of probiotics on PwP.

## MATERIALS AND METHODS

### Inclusion criteria and study selection

This systematic review was performed by two reviewers (J-H.C. and C-T.H.), and any disagreements were resolved after a panel discussion involving three reviewers (J-H.C., C-T.H., and T-W. H.). Eligible studies included are (1) interventional clinical trials (RCTs or non-RCTs) (2) clearly report patient inclusion and exclusion criteria (3) comparing the effects of probiotics on PwP (4) available data on GI symptoms including bowel movement and stool consistency. Studies were excluded if the intervention (1) did not include PwP (2) did not use probiotics as intervention (3) did not clearly report the effect on GI symptoms (4) was not published in English. This study is registered with PROSPERO (CRD42021262036).

### Literature search strategy

We searched for interventional clinical trials published before May 2022 in the PubMed, Embase, and Cochrane databases following the Preferred Reporting Items for Systematic Review and Meta-Analysis (PRISMA) guidelines [28]. The search keywords were as follows: {(Parkinson disease [Title/Abstract] OR Parkinson’s disease [Title/Abstract]) AND (probiotics [Title/Abstract] OR *Bifidobacterium* [Title/Abstract] OR *Lactobacillus* [Title/Abstract] OR *Enterococcus* [Title/Abstract] OR *Limosilactobacillus* [Title/Abstract]). Only studies published in English were included.

### Data extraction

Baseline and outcome data were independently retrieved by two reviewers (J-H.C. and C-T.H.). Furthermore, data on study designs, study population characteristics, inclusion and exclusion criteria, main outcomes and adverse events were extracted. Decisions recorded individually by the reviewers were compared, and disagreements were resolved by a third reviewer (T-W.H.).

### Outcomes

All outcomes of the included studies were summarized for the literature review. The primary outcome of the meta-analysis was the effect of probiotics on constipation, including bowel movement and stool consistency.

### Appraisal of methodological quality

Two reviewers (C-T.H. and J-H.C.) independently assessed the methodological quality of each study using the revised Risk of Bias (version 2.0) method for the RCTs [29] and Risk of Bias Assessment Tool for Systematic Reviews-I (ROBIS-I) for the non-RCT, as recommended by the Cochrane Collaboration [30]. The included RCTs were scored to determine whether they had a high, medium, or low overall risk of bias. The risk of bias was calculated through the assessment of five domains: bias resulting from the randomization process, bias resulting from deviation from intended interventions, bias resulting from missing outcome data, bias in the measurement of outcomes, and bias in the selection of reported results. Serious, moderate, or low overall risk among non-RCTs was assessed for seven domains: bias resulting from confounding, bias in selection of participants into the study, bias in classification of interventions, bias resulting from deviations from intended interventions, bias resulting from missing data, bias in measurement of outcomes, and bias in selection of the reported result.

### Statistical analysis

Data for the meta-analysis were entered and analyzed using Review Manager 5.4 (The Cochrane Collaboration, Oxford, England). A meta-analysis was performed following the PRISMA guidelines. The standard deviation was calculated using the provided confidence interval (CI) limits, standard errors, or interquartile ranges, where appropriate. The effect sizes of continuous outcomes were reported as the standardized mean difference (SMD). The precision of effect sizes was reported using a 95% CI. A pooled estimate of the weighted mean difference (WMD) was computed using the DerSimonian and Laird random-effects method. A statistically significant result was indicated by a *p* value of <0.05 or a 95% CI that did not include 1 in the relative risk ratio and 0 in the WMD estimation. Statistical heterogeneity and inconsistencies in treatment effects across the studies were evaluated using the Cochrane Q test and I^2^ statistic, respectively. Statistical significance was set at a *p* value of <0.10 for the Cochrane Q test. Statistical heterogeneity across the studies was assessed using the I^2^ statistic, which quantifies the proportion of total outcome variability across studies.

### Data availability statement (DAS)

The authors confirm that the data supporting the findings of this study are available within the article and its Supplementary Materials. These data were derived from the following resources available in the public domain.

## RESULTS

### Search results and study characteristics

**Figure 1:**
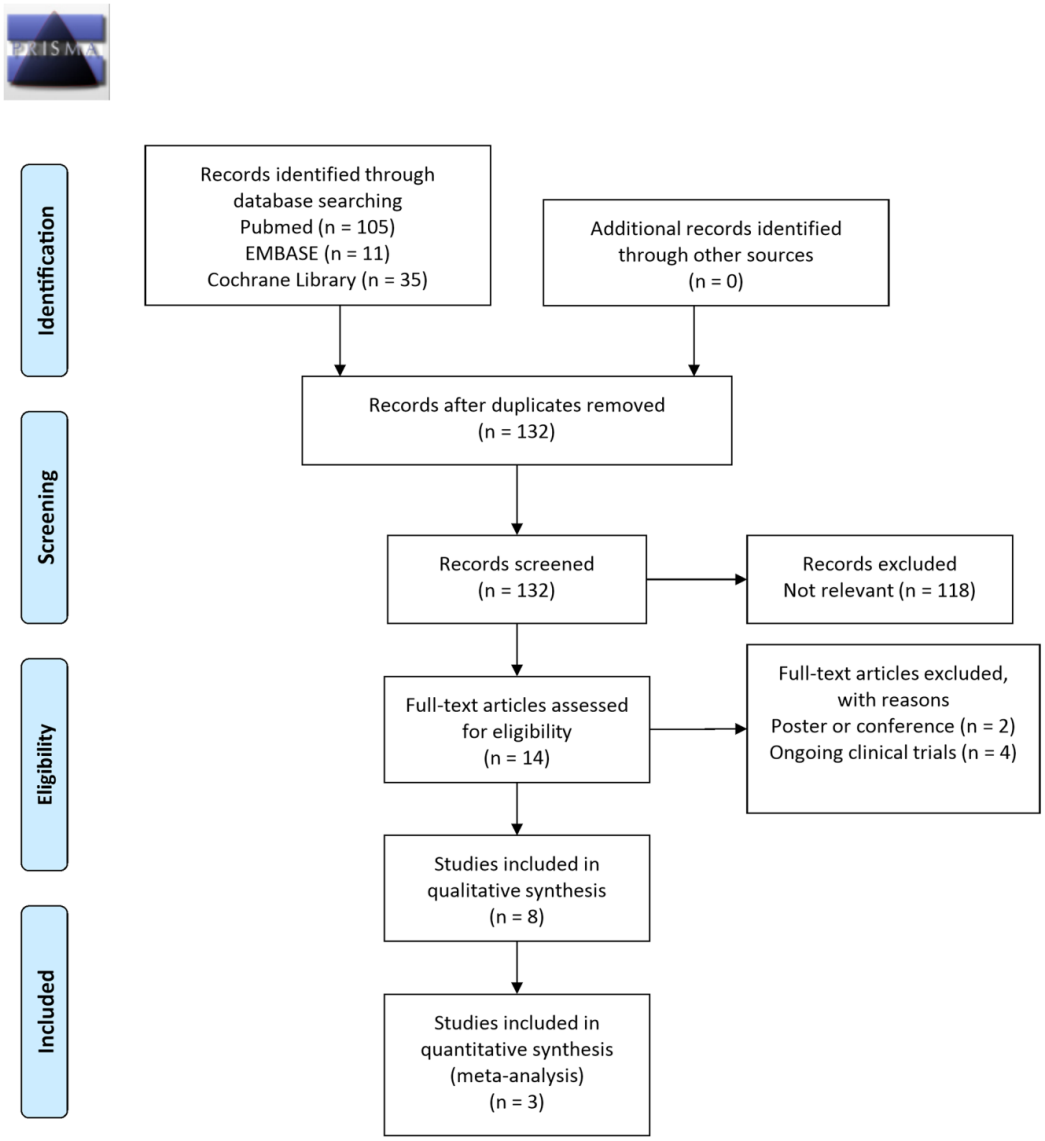
Study selection flowchart (RCT, randomized controlled trial).

Figure 1 displays the study selection flowchart. Our initial search yielded 151 studies, 19 of which were eliminated because of duplication. The remaining 132 studies were subjected to title and abstract screening, and 118 were excluded. The final 14 studies were entered in the full text review. Two studies were excluded because they were conference posters, and another four were excluded because they reported on ongoing clinical trials. The remaining eight clinical trials, including two open-label, single-arm studies [24, 31] and six eligible RCTs [23, 32–36], were included in the review. These studies were published between 2011 and 2021 and had sample sizes ranging from 25 to 120 PwP. All eight studies recruited PwP who had idiopathic PD diagnoses, and five of the studies specifically enrolled patients with functional constipation. The composition, amount, and treatment duration of probiotics varied, but Lactobacilli and Bifidobacterium were administered in most of the studies. Adverse events were reported in 6 studies, including bloating, abdominal distention, dizziness and lethargy. All of the adverse events were reversible and there was no serious adverse event noted (Table 1).

**Table 1:** Characteristics of the included clinical trials.

**Author [Year]/study design**	**Inclusion criteria**	**No. of patients (male, %)**	**Age/disease duration (years)**	**Probiotics: strains; CFU; treatment duration**	**Outcome**
Cassani [2011]/ Open-label, single-arm	PD with poor drug response constipation	40 (36, 90)	71.9/9.75	*Lactobacillus*; 6.5·10^9^; 5 weeks	BM, abdominal symptoms, stool consistency
Barichella [2016]/ RCT	PD with functional constipation	E: 80 (41, 51.2) C: 40 (24, 60.0)	E: 71.8/10.9 C: 69.5/9.6	*Streptococcus*, *Enterococcus*, *Lactobacillus*, *Bifidobacterium*; 250·10^9^; 4 weeks	BM, CBM, stool consistency
Georgescu [2016]/ RCT	PD with mid-to- moderate GI symptoms	probiotics: 20 (10, 50) trimebutine: 20 (7, 35)	probiotics: 69.8/7.05 trimebutine: 75.65/7.5	*Lactobacillus* and *Bifidobacterium*; 120 mg/day; 3 months	GI symptoms
Borzabadi [2018]/ RCT	PD	E: 25 (17, 68) C: 25 (16, 74)	E: 66.9/5.0 C: 66.7/5.4	*Lactobacillus* and *Bifidobacterium*; 8·10^9^; 12 weeks	Gene expression related to metabolism, biomarkers of inflammation and oxidative stress
Tamtaji [2018]/ RCT	PD	E:30 C:30	E: 68.2 C: 67.7	Lactobacillus and Bifidobacterium; 8·10^9^; 12 weeks	Metabolism profile
Ibrahim [2020]/ RCT	PD with functional constipation	E: 27 (16, 59.3) C: 28 (17, 60.7)	E: 69.0/6 C: 70.5/6.5	Lactobacillus Bifidobacterium; 30·10^9^, 8 weeks	BM, Garrigues Questionnaire, Gut transit time
Tan [2021]/ RCT	PD with functional constipation	E: 34 (20, 58.8) C: 38 (28, 73.7)	E: 70.9/9.7C: 68.6/10.1	*Enterococcus*, *Lactobacillus*, *Bifidobacterium*, *Limosilactobacillus*;10·10^9^; 4 weeks	BM, stool consistency, constipation severity score, quality of life
Lu [2021]/ open-label, single-arm	PD with OFF> 3 hours/day	25 (17, 68)	61.81/10.12	*Lactobacillus plantarum*; 3·10^9^; 12 weeks	UPDRS-III, PDQ-39, NMSS, BDI-II, PAC-SYM and PGI-C

BDI-II, Beck Depression Inventory-II; BM, bowel movement; CBM, complete bowel movement; C, control; CFU, colony-forming units; E: experimental; GI, gastrointestinal; PAC-SYM, Patient Assessment of Constipation Symptoms; PDQ-39, 39-item Parkinson’s Disease Questionnaire; PGI-C, Patient Global Impression of Change; RCT, randomized controlled trials; UPDRS-III, Unified Parkinson’s Disease Rating Scale part III.

### Risk of bias assessment

**Figure 2:**
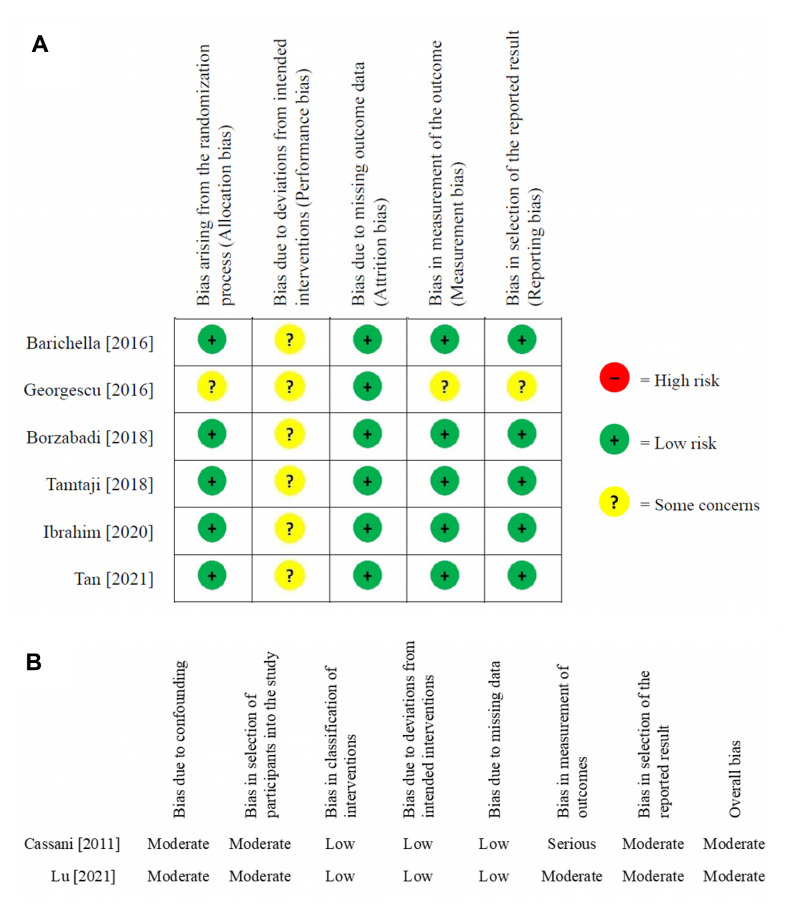
Risk of bias assessment (**A**) randomized controlled trials (**B**) nonrandomized controlled trials. green light with cross: low risk; yellow light with question mark: some concerns.

The results of risk of bias assessment are shown in Figure 2A, 2B. Overall, risk of bias was medium across the included RCT studies. All RCT studies had risk of some concerns regarding performance bias. One trial had another medium risk of allocation bias, measurement bias, and reporting bias. For non-RCT studies, both had a moderate overall risk of bias, especially on bias due to confounding, bias in selection of participants into the study, bias in measurement of outcomes, and bias in selection of the reported result.

### Review of include studies

**Figure 3:**
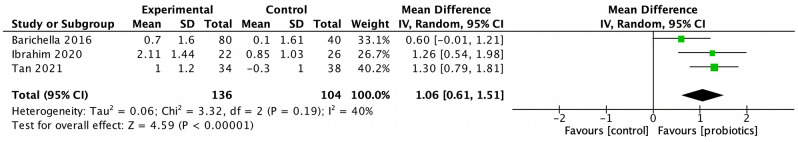
Effect of probiotics on frequency of bowel movements of people with Parkinson disease.

**Figure 4:**
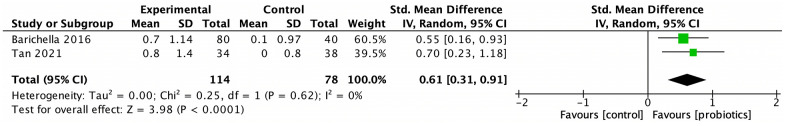
Effect of probiotics on the stool consistency of people with Parkinson disease.

Five studies investigated the effect of probiotics on GI symptoms, especially constipation, of PwP. Cassani et al. conducted an open-label, single-arm study to evaluate the benefits of probiotics on bowel movements, stool consistency, and clinical abdominal symptoms among PwP with constipation with poor drug response [24]. They found that 6-week probiotic treatment normalized the stool consistency and alleviated abdominal symptoms. Georgescu et al., however, conducted an RCT to compare probiotics with trimebutine, an intestinal spasmolytic agent, in PwP with mild-to-moderate GI symptoms [34]. Despite being inferior to trimebutine, probiotics significantly reduced the severity of abdominal pain and bloating when compared with data collected before treatment. However, because of the lack of a placebo control, this information can only be utilized as a single-arm, baseline-controlled trial to demonstrate the effect of probiotics. The remaining three RCTs, published between 2016 and 2021, evaluated the therapeutic benefit of probiotics on the GI symptoms of PwP. Two of the three studies were from Malaysia and added *Streptococcus* and *Enterococcus* to the *Lactobacillus* and *Bifidobacterium*–based regimen. The meta-analysis of these three studies demonstrated that the prescription of probiotics significantly increased weekly bowel movement (MD: 1.06, 95% CI: 0.61–1.51, *p* < 0.001, I^2^ = 40%; Figure 3). Stool consistency was evaluated in two studies, which presented a significant normalization of stool consistency (SMD: 0.61, 95% CI = 0.31–0.91, I^2^ = 0%; Figure 4). Ibrahim et al. also demonstrated probiotics’ significant reduction of gut transit time, which was measured using red carmine capsules to dye the stool.

Another two RCTs published by Iranian groups systematically investigated the influence of probiotics. These two RCTs recruited PwP and evaluated the changes in their metabolic profiles and gene expressions after 12 weeks of probiotics treatment. Tamtaji et al. argued that probiotic treatment resulted in a significant decrease in the Movement Disorder Society-Unified Parkinson Disease Rating Score (MDS-UPDRS) and a reduction of systemic inflammation (assessed by high-sensitivity C-reactive protein) and oxidative stress (assessed by malondialdehyde and enhanced glutathione) compared with placebo treatment [32]. Additionally, probiotic treatment also caused a statistically significant reduction in insulin levels and insulin resistance and a statistically significant rise in insulin sensitivity. Borzabadi et al. found that probiotic treatment downregulated the gene expression of proinflammatory cytokines (interleukin-1 and tumor necrosis factor-α) in peripheral blood mononuclear cells compared with controls [35]. Furthermore, probiotics upregulated the gene expression of the anti-inflammatory cytokine, transforming growth factor-β, and the metabolism regulator, peroxisome proliferator-activated receptor gamma. These two RCTs indicated that probiotics’ manipulation of gut microbiota may work not only on GI symptoms but also systemic pathology.

Last, Lu et al. published the latest, open-label, single-arm study that investigated the influence of probiotics on the motor fluctuation of PwP [31]. The prescribed probiotic was a single strain, *Lactobacillus plantarum*, and after 12 weeks of supplementation, UPDRS motor scores improved significantly in both the “OFF” and “ON” states, and the daily “OFF” period of time was reduced. However, no significant improvement occurred in the NMS of PwP.

Of the eight total studies, three studies mentioned adverse effects of probiotics. In general, these were tolerable and resolved after probiotic discontinuation. Tan et al. reported one case of lethargy after treatment with probiotics [36], and Ibrahim et al. reported two patients with abdominal symptoms and two with dizziness [33].

### Meta-analysis results for probiotics treatment

The meta-analysis of three studies demonstrated that the prescription of probiotics significantly increased weekly bowel movement (MD: 1.06, 95% CI: 0.61–1.51, p < 0.001, I2 = 40%; Figure 3). Stool consistency was evaluated in two studies, which presented a significant normalization of stool consistency (SMD: 0.61, 95% CI = 0.31–0.91, I2 = 0%; Figure 4).

## DISCUSSION

The present study conducted a literature review of interventional clinical trials to determine the effect of probiotics on PwP and performed a meta-analysis of RCTs to investigate the benefit of probiotics on GI symptoms, especially constipation, among PwP. In general, despite the heterogenicity of the bacterial strains and variations in the amount of probiotics and durations of the treatment regimes, probiotics were shown to significantly increase the frequency of bowel movements and normalize stool consistency among PwP. The side effects are generally tolerable. The present study provides evidence for the prescription of probiotics for PwP, especially for their GI symptoms.

The gut microbiota contains more genomes than any other body location [37]. Altered gut microbiota is associated with many health conditions and has a bidirectional causal relationship [38–40]. An association between the gut and PD has been suspected [22]. Constipation heralds the onset of PD motor symptoms decades ahead of time [41, 42], and the PD pathology, namely α-synuclein aggregation, may originate from the myenteric plexus in the intestine [43, 44]. PwP are also known to embrace gut dysbiosis [45, 46]. Although the intestines’ sterile condition attenuates PD pathology in clinical *in vivo* models [47], possible methods of manipulating the gut microbiota may only be possible by using prebiotics, probiotics, or symbiotics. According to the present review and meta-analysis, however, how probiotics affect PD-specific disease mechanisms, such as the degeneration of dopaminergic neurons and α-synuclein aggregation, has not been a research focus. Rather, the primary outcomes of most of the clinical trials were mainly GI symptoms, and only two studies from Iranian groups assessed the influence of oxidative stress and inflammation on patient metabolic profiles and gene expression. Metabolic syndrome, oxidative stress, and inflammation are contributors to neurodegeneration in PD [48, 49]. Neuroinflammation is one of the major pathogeneses of PD, and the activation of microglia was noted in post-mortem substantia nigra of PD brains [50]. Systemic inflammation also contributed to an increase in the risk of PD [51]. PwP exhibited elevated blood cytokine levels in serum and plasma extracellular vesicles compared with healthy controls [52, 53]. (Gut dysbiosis is a substantial risk factor of elevated systemic inflammation through the destruction of the intestinal epithelial membrane and the entrance of pathogens and toxins from the intestinal lumen into the systemic circulation [54]. On the hand, diabetes is a well-known risk factor for PD [55], and poor glucose control is a predictor of rapid disease progression [56]. Insulin resistance triggers the significant PD pathogeneses, namely mitochondrial dysfunction and α-synuclein accumulation, and neuronal insulin resistance is remarkable in PwP [57, 58]. Tamtaji et al. demonstrated that probiotic treatment resulted in a reduction in inflammation (assessed by a high-sensitivity C-reactive protein test) and oxidative stress (assessed by glutathione levels) and an increase in insulin sensitivity [32]. With regard to the disease course of PD, the short-term studies used in this meta-analysis cannot support a claim of a disease modification effect based on their short-term benefits. Further long-term studies are necessary to determine the effects of probiotic-related anti-inflammation, antioxidation, and antimetabolic syndrome on decelerating the neurodegenerative process of PD.

The gut microbiota plays an essential role in digestion and is strongly associated with health conditions of the GI system [59]. Probiotic supplementation, which introduces beneficial strains to the gut, may help restore the balance of the gut microbiota [60]. That *Lactobacilli* and *Bifidobacterium* in fermented foods relieve GI symptoms and promote bowel movements has been recognized for decades [61]. Studies have determined the abundance of *Lactobacilli* among PwP is greater than among controls [11, 46]. This suggests that subsequent investigations are needed to assess the ability of exogenous supplements of *Lactobacilli* to attenuate constipation and other GI symptoms among PwP. The present review and meta-analysis determined the consistent benefit of probiotic supplements containing *Lactobacilli* on the frequency of bowel movements and normalization of stool consistency. These results may instigate further investigations into whether the association between the greater abundance of *Lactobacilli* among PwP is a causal disease-pathognomonic relationship or constipation-related compensatory change. However, no sufficient evidence exists to clearly delineate this matter at this time.

The present study provided meta-analysis-based evidence for the benefit of probiotic therapy on constipation among PwP. The latest evidence-based medicine recommendations, based on the RCT published by Barichella et al., for managing constipation among PwP indicate that probiotics are “efficacious” and “clinically useful” [21, 23]. The present meta-analysis could further consolidate this concept after further RCTs are conducted and studied. Additionally, the present study also revealed a possible systemic effect, including the enhancement of probiotics on PD, which may lead to a possible disease modification effect or facilitate the body’s drug response through the manipulation of inflammation, oxidative stress, insulin sensitivity and metabolism.

However, the present study also involved certain limitations. First, only eight studies conducted in five countries, of which three were in Asia and two were in Europe, were included in this study. A person’s environment and diet greatly affect the gut microbiota [62]; therefore, the lack of studies from Africa, Oceania, and the Americas potentially limits the study’s global representation. Besides, the present review is limited to studies published in English, which means some studies may be omitted. Second, the present study could not determine whether the increase of *Lactobacilli* in PwP is detrimental or not. The regimens of probiotics in all studies contained *Lactobacilli*, despite the awareness that PwP already have more Lactobacilli than controls do [11]. Because the longest treatment period was only 12 weeks, probiotics’ long-term effects remain uncertain. Third, none of the studies investigated the change in stool short-chain fatty acids (SCFAs), which are potent antioxidants and one of the indicators of healthy gut microbiota [63]. Evidence of increased SCFAs in PwP’s stool after probiotic treatment would further enhance the argument for the beneficial effect of probiotic treatment on PwP. Fourth, the results of the meta-analysis were similar to each of enrolled study, which did not provide new information about the efficacy of probiotic on PwP. However, with the effort of meta-analysis, the findings were more consolidated and able to be listed in the future treatment suggestions of PwP. Last, the endogenous gut microbiota overwhelms supplementary probiotics, and the strict and competitive environment of the intestines reduces the possibility of long-lasting inhabitation of supplementary probiotics [64]. Future studies are suggested to compare the gut microbiota before and after treatment to demonstrate the direct effect of probiotics on PwP’s gut microbiota.

## CONCLUSIONS

This review and meta-analysis determined that probiotic treatments, mainly *Lactobacilli* and *Bifidobacterium*–based regimens, effectively alleviated constipation. Adverse effects are generally tolerable. However, considering the gut microbiota is highly associated with a person’s environment and diet, studies from other continents are required to establish the benefit of probiotics on constipation. Moreover, probiotic treatment is likely to affect the systemic inflammation and metabolism of PwP, but further studies are warranted to investigate the possibility of the disease modification effect on PD.
